# Surface Charge‐Determined Protein Coronas of Nanoparticles Control Endothelial Cells Uptake Under Low Magnitude Shear Stress

**DOI:** 10.1002/EXP.20240248

**Published:** 2026-02-11

**Authors:** Hongping Zhang, Shuang Zhao, Qianting Zhang, Chengchen Deng, Chuanrong Zhao, Xiangxiu Wang, Anna Malashicheva, Yi Wang, Juhui Qiu, Guixue Wang

**Affiliations:** ^1^ Key Laboratory for Biorheological Science and Technology of Ministry of Education State and Local Joint Engineering Laboratory For Vascular Implants Bioengineering College of Chongqing University Chongqing China; ^2^ JinFeng Laboratory Chongqing China; ^3^ Institute of Cytology Russian Academy of Science Saint Petersburg Russia; ^4^ School of Basic Medicine Chongqing Medical University Chongqing China

**Keywords:** apolipoprotein H, endothelial cells uptake, hemodynamics, protein corona, shear stress, surface charge of nanoparticles

## Abstract

Nanoparticles (NPs) are promising for atherosclerosis (AS) drug delivery, which involves exposure to low magnitude shear stress, including low shear stress and oscillatory shear stress. While NPs surface charge affects biodistribution and cellular uptake, its role in AS‐targeted accumulation remains unclear. In this study, positively charged NPs (pNPs), near‐electrically neutrally charged NPs (eNPs), and negatively charged NPs (nNPs) were employed to investigate their distribution and uptake in mice and endothelial cells (ECs). Here, we found that nNPs exhibited significantly greater accumulation and uptake by ECs at both atherosclerotic sites and regions subjected to low magnitude shear stress compared to pNPs and eNPs. Proteomic analysis revealed that the surface charge of the NPs profoundly influenced the composition of the protein corona. Specifically, nNPs adsorbed several orders of magnitude more apolipoprotein H (APOH) from serum than pNPs. Furthermore, low magnitude shear stress increased the levels of surface phospholipids, which are specific receptors for APOH, on ECs, thereby promoting the uptake of nNPs by ECs. In conclusion, our study uncovers a mechanism by which nNPs preferentially accumulate within atherosclerotic areas and uptake by ECs exposure to low magnitude shear stress, and provides insights for designing charge‐optimized NPs for cardiovascular drug delivery.

## Graphical abstract

To elucidate the mechanisms underlying NPs‐ECs interactions under hemodynamic conditions, our study reveals that low magnitude shear stress (LSS and OSS) enhances the uptake of nNPs by ECs, which exhibit a pronounced tendency to accumulate at atherosclerotic plaque sites in ApoE^−/−^ mice. The surface of these nNPs adsorbs substantial amounts of APOH, forming a protein corona. Furthermore, low magnitude shear stress upregulates the surface phospholipids content on ECs, thereby promoting their selective interaction with APOH.

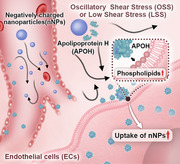

## 1 Introduction

Nanoparticles (NPs)‐based drug delivery systems have emerged as promising platforms for delivering therapeutic drugs to specific disease sites and minimizing off‐target organ toxicity [1, 2, 3, 4, 5]. The design of NPs with adjustable shapes, sizes, flexibilities, and surface charges is essential for modulating the interaction between NPs and cells, ultimately influencing the fate of NPs in vivo and their therapeutic effects [1, 6, 7, 8]. Among these parameters, the surface charge of NPs plays a critical role in their circulation and biological distribution [9]. During circulation in the bloodstream, NPs adsorb plasma proteins, forming a protein corona (PC) on their surface, which influences their biodistribution, bioavailability, biotransformation in vivo [10, 11], and cellular uptake of NPs [12, 13]. However, it remains unclear whether the surface charge of NPs can affect the protein composition and content of the adsorbed PC and subsequently influence the uptake of NPs by endothelial cells (ECs). Therefore, elucidating the association between the NPs surface charge and PC formation will facilitate the design of NPs to increase delivery efficiency.

Atherosclerotic cardiovascular disease is one of the leading causes of mortality worldwide [14]. It predominantly occurs in areas with low magnitude shear stress [15], including low shear stress (LSS, <5 dyne cm^−2^) and oscillatory shear stress (OSS, 0.5 ± 4 dyne cm^−2^). Therefore, hemodynamic force plays a crucial role in the development of atherosclerosis (AS) [16, 17, 18]. ECs, which are located in the inner layer of blood vessels and are directly exposed to blood flow, can rapidly respond to changes in shear stress, influencing their function and behavior. For example, ECs in arterial branches and curvatures with OSS exhibit a proinflammatory phenotype, leading to an increased oxidative stress response, mitochondrial dysfunction, and metabolic abnormalities, thereby worsening AS progression [19]. On the other hand, normal shear stress (NSS, 12 dyne cm^−2^) with a unidirectional shear direction in relatively straight arteries is protective against AS [20]. Our recent studies have proposed that ECs are a type of nonprofessional phagocytic cells and that their uptake behavior is influenced by shear stress [21, 22, 23]. However, how the surface charge of NPs mediates the phagocytosis of ECs under shear stress has not been explored.

**FIGURE 1:**
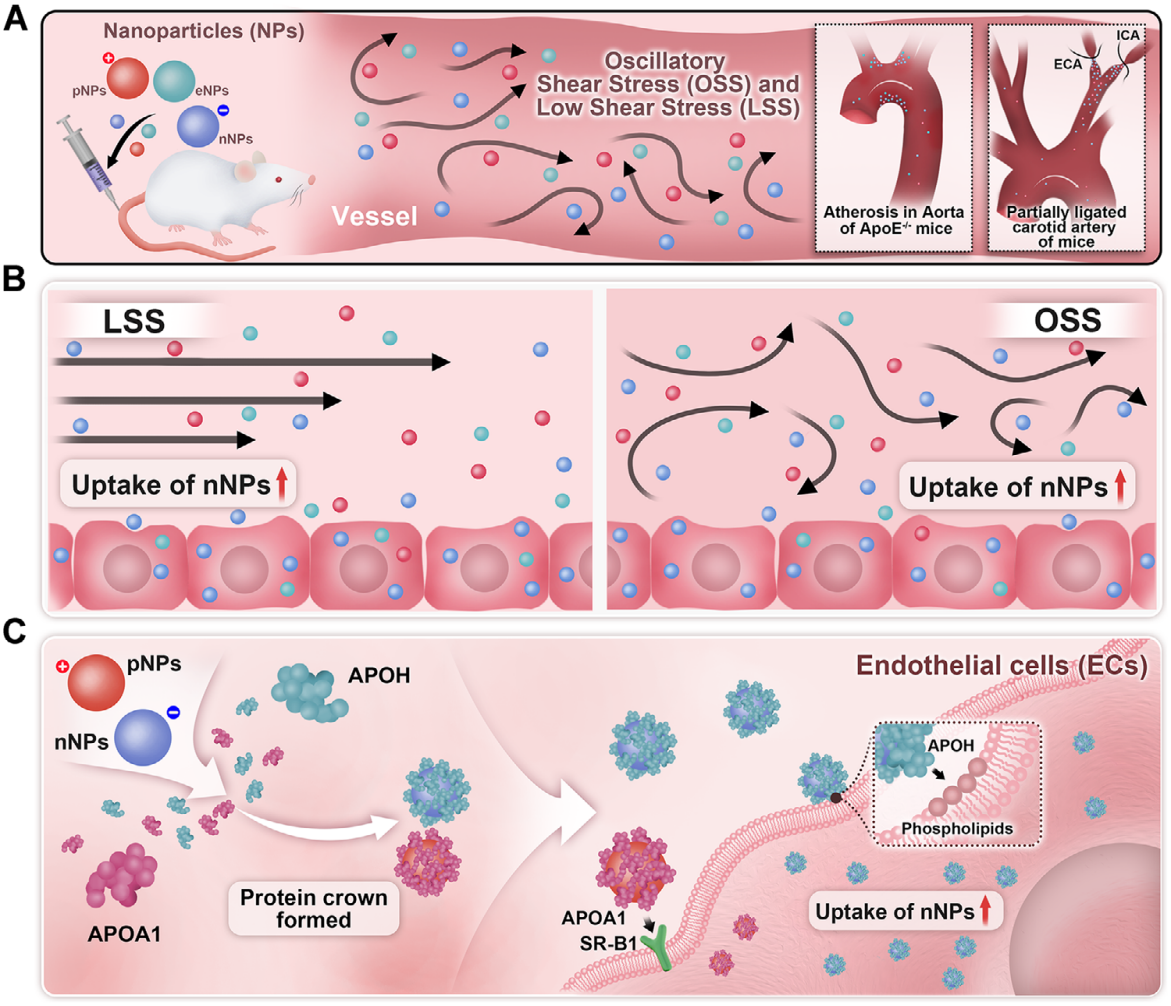
Illustrations showing that nNPs adsorbed APOH to increase their uptake by ECs under low magnitude shear stress (LSS and OSS). (A) Low magnitude shear stress promotes the uptake of nNPs by ECs in vivo. (B,C) APOH partially mediates the low magnitude shear stress‐induced increase in nNPs uptake by ECs.

In this study, we aimed to investigate the influence and biomechanical mechanisms of NPs’ surface charge on NPs distribution in AS mice and NPs uptake by ECs under shear stress. We found that, compared with those of positively charged NPs (pNPs) and near‐electrically neutrally charged NPs (eNPs), the uptake of negatively charged NPs (nNPs) by ECs under low magnitude shear stress (LSS and OSS) was significantly increased both in vitro and in vivo. Furthermore, nNPs did not cause strong inflammatory responses in apolipoprotein E^−/−^ (ApoE^−/−^) mice. Our results revealed that different surface charges of the NPs influenced the adsorption of PC. Specifically, nNPs adsorbed hundreds of times more apolipoprotein H (APOH) than pNPs did. Additionally, low magnitude shear stress increased the levels of phospholipids on the surface of ECs, which serve as specific binding sites for APOH. This may be the primary reason why nNPs exhibited increased accumulation in AS lesion areas and increased uptake by ECs under low magnitude shear stress (Figure 1). In conclusion, our study uncovers a mechanism by which nNPs preferentially accumulate within atherosclerotic areas and uptake by ECs, highlighting their potential as effective nanocarriers for targeted AS therapy.

## 2 Results

### 2.1 nNPs Tend to Accumulate at AS Lesion Sites in ApoE^−/−^ Mice and at OSS Regions In Vivo

To investigate the effects of the surface charge of NPs on ECs uptake in vivo and in vitro, pNPs, eNPs and nNPs were obtained and characterized. First, transmission electron microscopy (TEM) revealed that pNPs, eNPs and nNPs were spherical, with average diameters of 102, 104, and 103 nm, respectively (Figure S1A). Moreover, there was no significant difference in size among pNPs, eNPs, and nNPs (Figure S1B). The size distributions pNPs, eNPs, and nNPs were further tested by dynamic light scattering (Figure S1C). The hydrodynamic diameters of the pNPs, eNPs, and nNPs in distilled water were approximately 126, 109, and 108 nm, respectively (Figure S1D). The polymer dispersity index of pNPs, eNPs, and nNPs were approximately 0.072, 0.032, and 0.019, respectively. The ζ‐potentials of pNPs, eNPs, and nNPs in distilled water were measured as approximately 40.7, 6.8, and −40.1 mV, respectively (Figure S1E). Second, the three NPs exhibited coincident fluorescence spectra (Figure S2). Moreover, there was no significant difference in the fluorescence intensity among the three types of NPs at the same concentration (10 µg mL^−1^) (Figure S3). These results indicated that the three types of NPs prepared from the same materials have similar particle sizes and no significant difference in the fluorescence intensity at the same concentration.

**FIGURE 2:**
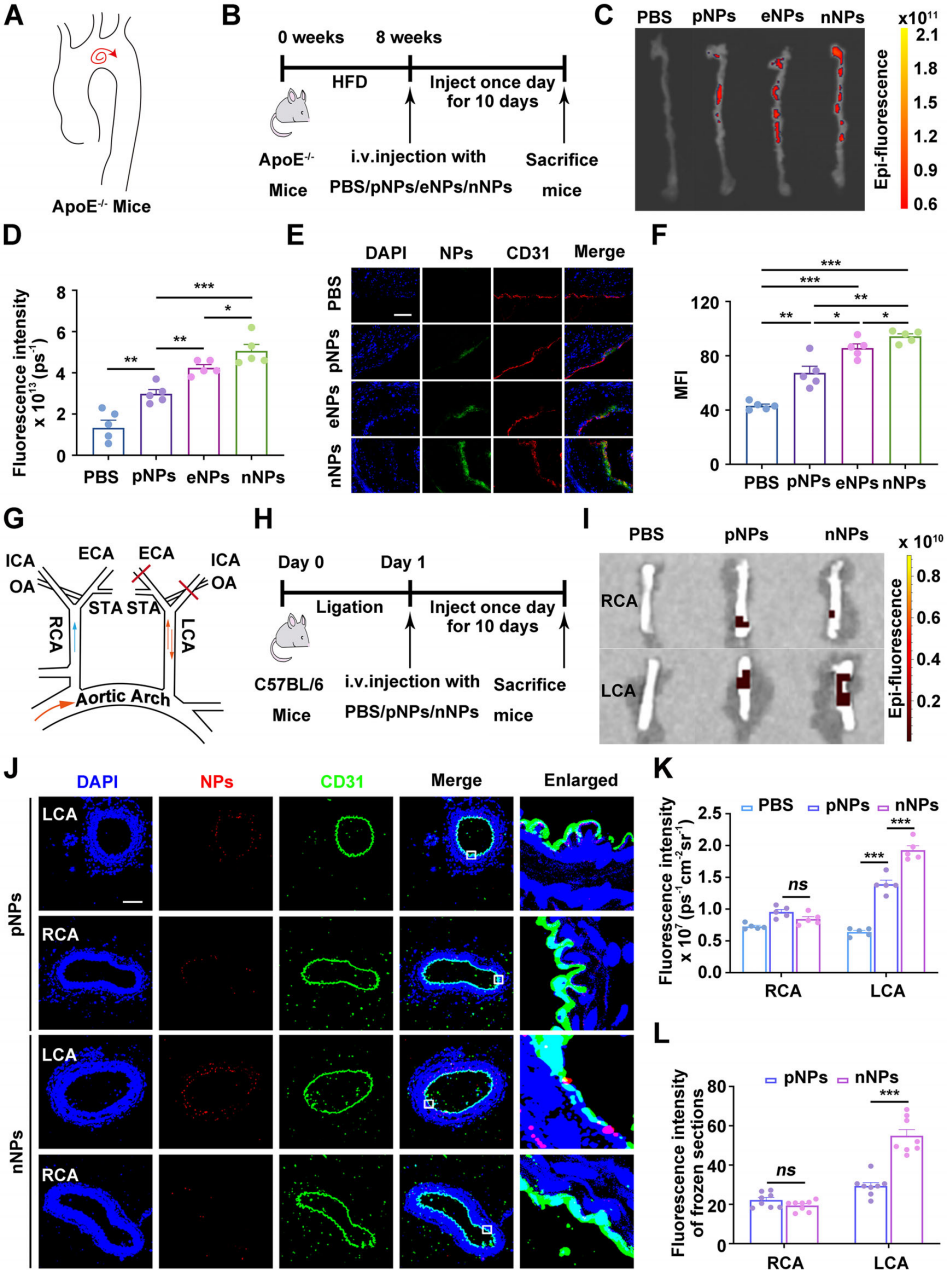
nNPs tend to accumulate at the AS lesion sites in ApoE^−/−^ mice and at OSS regions in vivo. (A) Diagram illustrating disturbed flow in the aortas of ApoE^−/−^ mice. (B) Schematic of the construction process of the AS mice models, and the injection time of the NPs. HFD, high‐fat diet. (C) Aorta imaging via the small animal optical system. (D) Quantitative analysis of fluorescence intensity in the aortas of mice subjected to different treatments (*n*  =  5). (E) CD31 immunofluorescence staining of the aortic arch root. DAPI, 4′,6‐diamidino‐2‐phenylindole. Scale bar: 100 µm. (F) Quantitative analysis of the fluorescence intensity of the NPs in the aortic arch roots of the different groups (*n*  =  5). MFI, mean fluorescence intensity. (G) Schematic diagram of the ligation procedure. The LCA of C57BL/6 mice was ligated, and the mice were injected daily with PBS, pNPs, or nNPs for 10 days. External carotid artery (ECA), internal carotid artery (ICA), occipital artery (OA), and superior thyroid artery (STA). (H) Schematic of the injection time of NPs in partial carotid artery ligation mice models. (I) Imaging of the LCA and RCA via a small animal optical system. (J) CD31 immunofluorescence staining of the carotid artery. Scale bar: 100 µm. (K) Quantitative analysis of fluorescence intensity in the carotid artery of mice subjected to different treatments (*n*  =  5). (L) Quantitative analysis of the fluorescence intensity of NPs in the carotid artery from the different groups (*n*  =  8). “*ns*” stands for non‐significance, ^*^ *p* < 0.05, ^**^ *p* < 0.01, ^***^ *p* < 0.001.

The cytotoxicity of the three types of NPs was evaluated. When the treatment concentration was no higher than 10 µg mL^−1^, none of the three types of NPs affected cell viability, which was chosen as the subsequent treatment concentration for the cell experiments (Figure S4A–C). The accumulation of pNPs, eNPs, and nNPs at the AS lesion sites was subsequently detected in AS mice models (Figure 2A,B). The fluorescence signal of nNPs in the aorta was significantly stronger than that of pNPs and eNPs, indicating a greater tendency for nNPs to accumulate in the OSS area (Figure 2C,D). Additionally, cluster of differentiation 31 (CD31) immunofluorescence staining of aortic arch root sections revealed greater uptake of nNPs by ECs than that of pNPs and eNPs (Figure 2E,F). These findings suggest that nNPs are more prone to accumulate in the atherosclerotic plaque area and be phagocytosed by ECs compared to pNPs and eNPs. Furthermore, pNPs, eNPs, and nNPs were found to be predominantly distributed in the liver (Figure S5A,B), as the liver is the main organ responsible for NPs elimination [24, 25]. We also used positively charged gold NPs (pGNPs) and negatively charged gold NPs (nGNPs) to investigate the accumulation of NPs in atherosclerotic plaque areas. The pGNPs and nGNPs have similar particle sizes and typical positive and negative charges (Figure S6A–E). The inductively coupled plasma mass spectrometry analysis revealed that the content of nGNPs in the aorta of the AS mice model (approximately 780 µg kg^−1^) was significantly higher than that of pGNPs (approximately 151 µg kg^−1^) (Figure S6F).

AS frequently localizes to regions of OSS [26, 27, 28]. To directly evaluate the effects of OSS on the uptake of pNPs and nNPs by ECs in vivo, we employed partial carotid artery ligation mice models [29, 30]. The left carotid artery (LCA) was subjected to ligation, resulting in OSS, whereas the non‐ligated right carotid artery (RCA) maintained laminar flow with NSS [31, 32]. The ligation process and tail vein injection are depicted in Figure 2G,H. The fluorescence signals of pNPs and nNPs in the LCA and RCA were observed via a small animal optical imaging system. As shown in Figure 2I,K, the accumulation of nNPs in the LCA was significantly greater than that of pNPs. However, there was no significant difference between nNPs and pNPs in the RCA. To further demonstrate the uptake of pNPs and nNPs by ECs, immunofluorescence staining of frozen sections from the LCA and RCA was performed. As shown in Figure 2J,L, ECs in the LCA engulfed more nNPs than pNPs. These findings highlight the critical role of low magnitude shear stress in regulating the uptake of nNPs and pNPs by ECs in vivo, and its ability to promote the internalization of nNPs by ECs.

### 2.2 LSS Increases the Uptake of nNPs by ECs In Vitro

**FIGURE 3:**
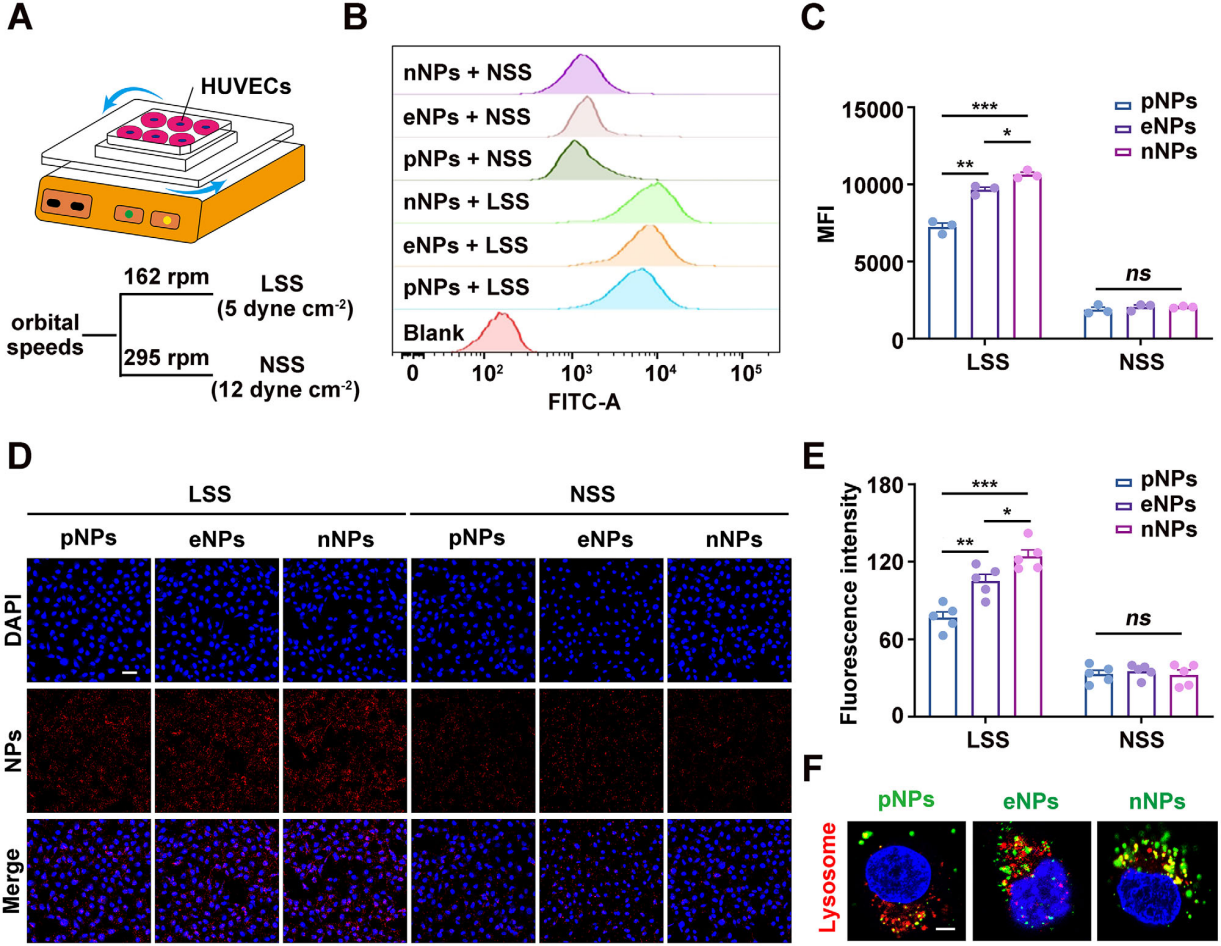
Uptake of pNPs, eNPs, and nNPs by HUVECs in vitro. (A) Schematic diagram of the orbital shaker system. (B) Flow cytometry analysis of NPs uptake by HUVECs after 3 h of treatment with LSS or NSS. (C) Quantitative analysis of B (*n*  =  3). (D) Laser confocal images of NPs taken up by HUVECs under different shear stress for 3 h. Scale bar: 50 µm. (E) Quantitative analysis of D (*n*  =  5). (F) HUVECs were treated with pNPs, eNPs or nNPs for 3 h and stained with LysoTracker Red. Scale bar: 5 µm. “*ns*” stands for non‐significance, ^*^ *p* < 0.05, ^**^ *p* < 0.01, ^***^ *p* < 0.001.

To determine whether shear stress influences the uptake of pNPs, eNPs and nNPs by ECs, we used an orbital shaker system to simulate LSS and NSS (Figure 3A) [21, 33]. Flow cytometry analysis revealed an increase in the uptake of nNPs by human umbilical vein endothelial cells (HUVECs) compared with that of pNPs and eNPs under LSS, whereas there was no significant difference under NSS (Figure 3B,C). Laser confocal microscopy further revealed that, under LSS, HUVECs internalized more nNPs than pNPs and eNPs did (Figure 3D,E). Additionally, lysosome staining indicated that a portion of the NPs did not colocalize with lysosomes after their cellular uptake (Figure 3F). These results indicate that low magnitude shear stress significantly enhances the uptake of pNPs, eNPs, and particularly nNPs by ECs.

### 2.3 Proteomic Fingerprints of PC Modulated by the Surface Charge of NPs

**FIGURE 4:**
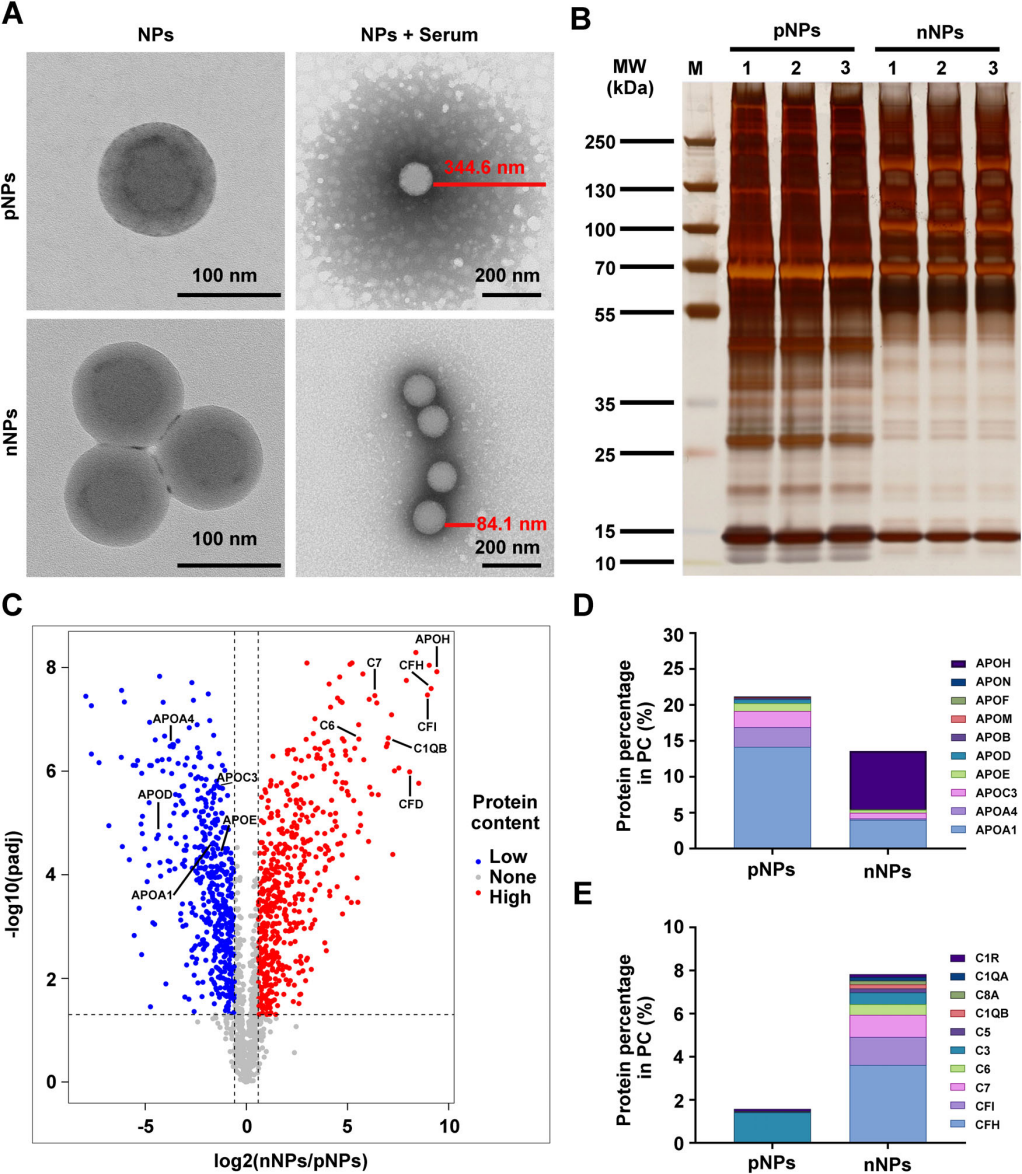
Proteomic fingerprints of PC modulated by the surface charge of the NPs. (A) TEM images of the NPs before and after incubation with 10% FBS at 37°C for 3 h. (B) Silver‐stained SDS‐PAGE gel of PC on pNPs and nNPs. MW, molecular weight (kDa). (C) Volcano plot of the protein fold change (log2(nNPs/pNPs)) plotted against the statistical significance (−log10(padj)). Proteins with increased content are shown in red and proteins with decreased content are shown in blue. The horizontal dashed line represents *p* = 0.05. The vertical left dashed line represents a fold change  =  2/3, and the vertical right dashed line represents a fold change  =  1.5. (D,E) Percentages of the identified apolipoproteins (D) and complement proteins (E) in PC adsorbed on pNPs and nNPs.

Our previous research indicated that NPs tend to accumulate in the OSS region, where their uptake by ECs is influenced by OSS [21]. In this work, we demonstrated that ECs engulf more nNPs than pNPs in the low magnitude shear stress region. Therefore, we investigated the charge‐dependent uptake mechanism by ECs under low magnitude shear stress. In biological fluid, NPs form a protein‐rich layer known as PC [34], which plays a pivotal role in determining the biological fate and behavior of NPs in vivo. Therefore, we hypothesize that differences in the composition of PC adsorbed on pNPs and nNPs might mediate their differential uptake by ECs. We analyzed the morphologies of pNPs and nNPs before and after incubation with 10% fetal bovine serum (FBS) via TEM. TEM analysis revealed a distinct PC on the surface of the NPs after incubation in 10% FBS. Notably, the thickness of the PC was approximately 345 nm on the pNPs, whereas it was substantially thinner (84 nm) on the nNPs (Figure 4A).

To characterize the protein adsorption profiles of PC, we analyzed the protein content adsorbed by the NPs and found that pNPs adsorbed more proteins than nNPs did, indicating strong interactions between the pNPs and FBS proteins (Figure S7A). Sodium dodecyl sulfate‐polyacrylamide gel electrophoresis (SDS‐PAGE) analysis of the PC formed on the NPs confirmed that different types of proteins adsorb onto pNPs and nNPs (Figure 4B). Notably, pNPs tended to adsorb proteins with molecular weights ranging from 25–70 kDa, whereas nNPs tended to adsorb proteins with molecular weights ranging from 50–200 kDa. To gain further insight into the protein fingerprints of the PC formed on pNPs and nNPs, proteomic analysis was performed. Compared with those adsorbed by pNPs, the PC adsorbed by nNPs contained 543 proteins with increased content and 503 proteins with decreased content (Figure S7B). Among the proteins with significant differences, apolipoprotein A1 (APOA1) was the most adsorbed protein on pNPs, with APOH being the most adsorbed protein on nNPs. Notably, nNPs adsorbed hundreds of times more APOH than pNPs did (Tables S1 and S2). Electrophoresis and mass spectrometry also supported these findings, revealing a prominent band at 30 kDa (APOA1) in pNPs and a prominent band at 55 kDa (APOH) in nNPs. By comparing the abundant proteins in the two groups, we observed that pNPs presented more apolipoproteins (e.g., APOA1) (Figure 4C,D), whereas nNPs presented more complement proteins (e.g., complement factor H) (Figure 4C,E). In summary, our results suggest that the significant differences in the proteins adsorbed on the surfaces of pNPs and nNPs may mediate their uptake by ECs under shear stress.

### 2.4 APOH Is Partially Responsible for the Low Magnitude Shear Stress‐Induced Increase in nNPs Uptake by ECs

**FIGURE 5:**
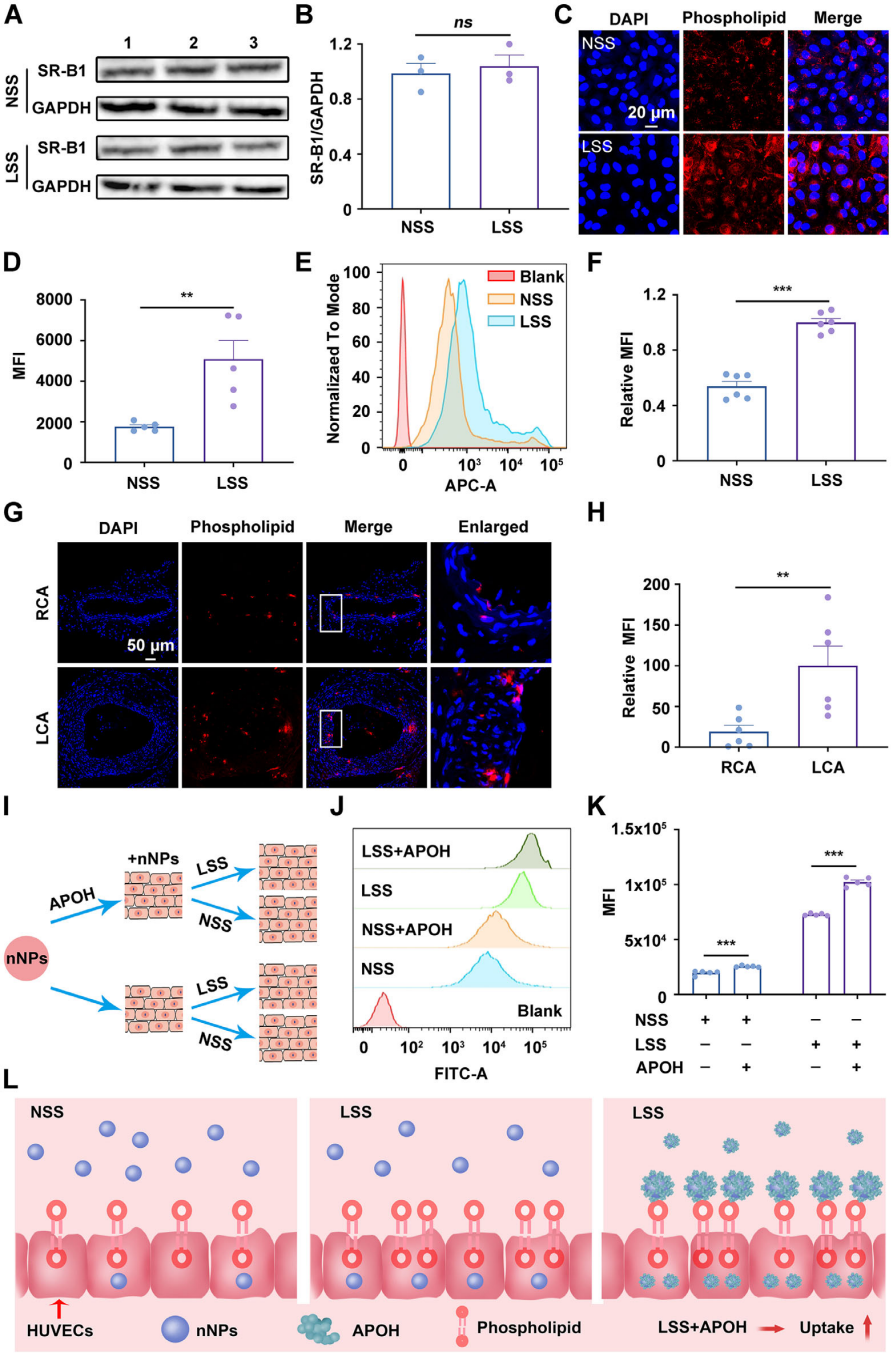
APOH is partially responsible for low magnitude shear stress‐induced increase in nNPs uptake by HUVECs. (A) Immunoblotting of SR‐B1 protein in HUVECs treated with LSS or NSS for 3 h (*n*  =  3). (B) The relative intensity of the SR‐B1 in HUVECs treated with LSS or NSS for 3 h (*n*  =  3). (C,D) Confocal imaging analyses of phospholipids in HUVECs treated with LSS or NSS for 3 h in vitro (*n*  =  5). Nuclei were stained with DAPI (blue), and the pseudo‐color of the phospholipids is shown in red. Scale bar: 20 µm. (E,F) Flow cytometry evaluation of phospholipids in HUVECs treated with LSS or NSS for 3 h (*n*  =  6). (G,H) Confocal imaging analyses of phospholipids in vivo (*n*  =  6). Nuclei were stained with DAPI (blue), and the pseudo‐color of the phospholipids is shown in red. Scale bar: 50 µm. (I) Schematic diagram of HUVECs uptake after different treatments. (J,K) Flow cytometry analysis of the uptake of nNPs pretreated with APOH by HUVECs for 3 h in RPMI‐1640 medium without 10% FBS (*n*  =  5). (L) nNPs were pretreated with APOH to investigate the effect of APOH on the uptake of nNPs in RPMI‐1640 medium without 10% FBS. Compared with NSS, LSS increased the phospholipid content at the binding sites of APOH on the surface of HUVECs, thereby enhancing nNPs uptake by HUVECs. “*ns*” stands for non‐significance, ^**^ *p* < 0.01, ^***^ *p* < 0.001.

Proteomic analysis revealed that nNPs adsorbed more APOH and that pNPs adsorbed more APOA1 from the serum, which suggests that these proteins may play a vital role in the uptake process of ECs under low magnitude shear stress. The phospholipids and scavenger receptor class B member 1 (SR‐B1) on ECs are the receptors of APOH and APOA1, respectively [35, 36, 37]. Therefore, we tested the expression of phospholipids and SR‐B1 on the ECs subjected to LSS or NSS. When ECs were exposed to LSS or NSS, we observed no significant difference in the expression of SR‐B1 (Figure 5A,B). However, confocal microscopy revealed that, compared with NSS, LSS significantly increased the phospholipid content on the EC membrane (Figure 5C,D). Flow cytometry analysis also confirmed a twofold increase in the content of phospholipids on ECs under LSS compared with NSS (Figure 5E,F). Additionally, in the partial carotid ligation mice models, the content of phospholipids on ECs in the LCA was significantly increased than that in the RCA (Figure 5G,H). Collectively, these results indicate that low magnitude shear stress can increase the content of phospholipids on ECs, potentially contributing to the nNPs increased uptake. To further explore whether APOH mediates the uptake of nNPs by HUVECs, we treated HUVECs as shown in Figure 5I. As shown in Figure 5J,K, preincubating nNPs with APOH increased their uptake by HUVECs under LSS. Additionally, preincubating nNPs with APOH alleviated the NSS‐induced inhibition of uptake. Overall, these findings suggest that low magnitude shear stress may promote the uptake of nNPs by increasing the levels of phospholipids on the ECs membrane (Figure 5L).

### 2.5 Side Effects Assessment of pNPs, eNPs and nNPs in ApoE^−/−^ Mice

**FIGURE 6:**
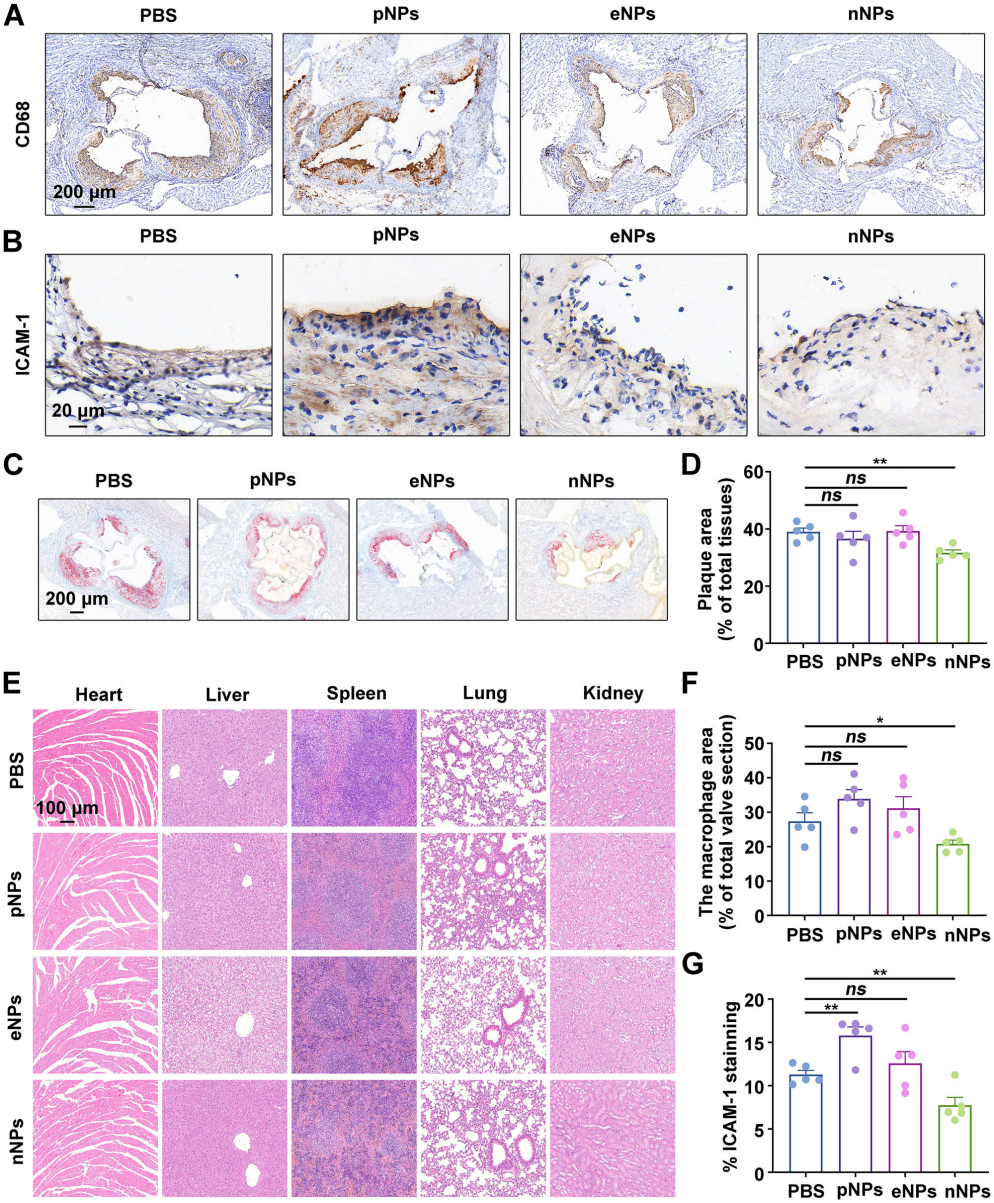
Side effects of charge‐dependent NPs in ApoE^−/−^ mice. (A) Representative images of immunohistochemical staining for CD68 in the root of the aortic arch after different treatments. Scale bar: 200 µm. (B) Representative images of immunohistochemical staining with antibodies against ICAM‐1 in the root of the aortic arch after different treatments. Scale bar: 20 µm. (C) Oil red O staining of the root of the aortic arch. Scale bar: 200 µm. (D) Quantitative analysis of C (*n*  =  5). (E) HE‐stained images of the main organs of the mice after different treatments. Scale bar: 100 µm. (F) Quantitative analysis of A (*n*  =  5). (G) Quantitative analysis of B (*n*  =  5). “*ns*” stands for non‐significance, ^*^ *p* < 0.05, ^**^ *p* < 0.01.

A major advantage of NPs‐mediated drug delivery systems is their ability to increase the biosafety of therapeutic agents administered systemically [38, 39]. Therefore, the safety of the NPs themselves is also very important. We further examined the side effects of pNPs, eNPs, and nNPs, especially in atherosclerotic plaque areas. First, we analyzed the expression of the cluster of differentiation 68 (CD68), a marker of macrophages, in the aortic arch roots of ApoE^−/−^ mice. As shown in Figure 6A,F, nNPs did not exacerbate macrophage accumulation compared to PBS. Second, intercellular cell adhesion molecule‐1 (ICAM‐1), an adhesion molecule, is overexpressed during ECs dysfunction, thereby enhancing peripheral monocyte adhesion. To investigate whether pNPs, eNPs, and nNPs can induce inflammatory responses in ECs, we performed immunohistochemical staining for ICAM‐1. Treatment with nNPs did not exacerbate ICAM1 expression compared to PBS (Figure 6B,G). These results indicated that nNPs did not cause inflammatory responses in atherosclerotic plaque areas. Furthermore, nNPs treatment showed minimal lipid deposition at the root of the aortic arch (Figure 6C,D). HE staining of the heart, liver, spleen, lung, and kidney of ApoE^−/−^ mice revealed no noticeable lesions or abnormalities after the different treatments (Figure 6E). The complete routine blood reports and liver function test results are shown in Tables S3 and S4. These results indicate that nNPs do not cause inflammatory responses in atherosclerotic plaque areas.

## 3 Discussion

ECs are located in the inner surface of blood vessels and play a critical role in maintaining vascular integrity and homeostasis. Because they are in direct contact with blood flow, ECs can respond swiftly to mechanical changes, thereby regulating their function. The surface charge of NPs affects their distribution and metabolism in organisms. Previous studies have shown that cyclic stretching influences the uptake of NPs with different surface charges in A549 cells in vitro, leading to a blebbing morphology and the activation of apoptotic signaling when combined with pNPs [40]. In this work, we aimed to investigate the effect of the surface charge of NPs on ECs uptake under shear stress. Surprisingly, we observed that, compared with pNPs and eNPs, low magnitude shear stress promoted the uptake of nNPs by ECs both in vitro and in vivo. Proteomic analysis revealed that nNPs adsorbed more APOH, whereas pNPs adsorbed more APOA1 from the serum. Mechanistically, the uptake of APOH‐bound nNPs by ECs depends on increased phospholipids on cell membranes induced by low magnitude shear stress. Furthermore, in vivo experiments revealed that nNPs did not cause inflammatory responses in atherosclerotic plaque areas. These findings suggest that nNPs may be more suitable for nanomedicine delivery systems in the cardiovascular field.

The biodistribution and cellular uptake of NPs can significantly impact their therapeutic efficacy and side effects in vivo [41]. Although the interaction of NPs with cells and their distribution in the circulatory system have been extensively studied [42], there is a lack of research on the uptake of NPs with different surface charges by ECs under varying shear stress. To address this gap, we employed AS mice models and partial carotid artery ligation mice models to investigate the uptake of pNPs, eNPs, and nNPs by ECs in vivo. In AS mice models, we confirmed that nNPs had a greater tendency to accumulate in lesion areas than pNPs and eNPs. Furthermore, in the partial carotid artery ligation mice models [43], which produce OSS in the LCA, we observed greater uptake of nNPs than of pNPs by ECs in the LCA. To further investigate the uptake of pNPs, eNPs and nNPs by ECs under LSS, we conducted in vitro studies. Interestingly, we found that LSS promoted the uptake of nNPs by ECs. In summary, our findings demonstrated that nNPs were preferentially internalized by ECs over pNPs and eNPs under low magnitude shear stress. Therefore, the surface charge of NPs plays a significant role in influencing their uptake by ECs, and these findings can guide the development of NPs‐based delivery systems for AS therapy.

Another critical aspect of NPs interactions is the inevitable binding of proteins in biological fluids, which leads to the formation of a PC on their surface. However, the underlying mechanism of how the PC influences the uptake of NPs by ECs under low magnitude shear stress remains unclear. In this study, the types and amounts of proteins adsorbed on pNPs and nNPs were investigated. We found that pNPs adsorbed more proteins than nNPs did. Moreover, nNPs adsorbed more APOH, whereas pNPs adsorbed more APOA1 from the serum. Additionally, we demonstrated that low magnitude shear stress could increase the uptake of nNPs by ECs by increasing the phospholipid content of the cell membrane. Preincubation of nNPs with APOH also increased their uptake by ECs under both LSS and NSS. Therefore, the phospholipid content on the EC membrane played an important role in the increased uptake of APOH‐adsorbed nNPs. Given the increased levels of APOH in the plasma of cardiovascular disease patients [44], the use of nNPs as a delivery system may be a highly promising therapeutic strategy for cardiovascular diseases, which tend to occur at sites with abnormal shear stress.

Considering the crucial role of nanomedicine in cardiovascular applications, it is vital to ensure that NPs do not aggravate atherosclerosis. Therefore, we evaluated the expression of CD68 and ICAM‐1 in atherosclerotic plaque areas. Compared with PBS, nNPs did not promote macrophage accumulation in AS areas or ICAM1 expression on ECs. This work revealed the associations among the surface charge of NPs, the PC, and ECs phagocytosis under shear stress. These findings have significant implications for the development and application of nanomedicine delivery systems, particularly in the field of cardiovascular medicine.

## 4 Conclusion

In this study, pNPs, eNPs and nNPs were used to investigate the influence of surface charge on NPs uptake by ECs under low magnitude shear stress (LSS and OSS). Compared with pNPs and eNPs, low magnitude shear stress increased the uptake of nNPs by ECs both in vitro and in vivo. Additionally, nNPs are preferentially phagocytosed by ECs in atherosclerotic plaque areas. Through proteomics analysis, we identified APOH as the main differentially adsorbed protein on the surface of nNPs compared with pNPs, which can interact with the increased phospholipid content on the ECs membrane under low magnitude shear stress. This interaction accelerates the uptake of nNPs by ECs under low magnitude shear stress. Furthermore, compared with PBS, the nNPs did not enhance macrophage accumulation or ICAM1 expression in AS areas. In general, this work sheds light on biomechanics, the surface charge of NPs, and PC‐mediated ECs uptake, which will accelerate the development of nanomedicine delivery strategies for cardiovascular diseases.

## Author Contributions

Hongping Zhang, Yi Wang, Juhui Qiu, and Guixue Wang, designed the research, conducted the experiments, analyzed the data and wrote the original draft. Shuang Zhao, Qianting Zhang, and Chengchen Deng conducted the experiments and analyzed the data. Chuanrong Zhao and Xiangxiu Wang conducted the analyses. Hongping Zhang, Anna Malashicheva, Yi Wang, Juhui Qiu, and Guixue Wang wrote and edited the manuscript. All the authors contributed to the revision of the manuscript and agreed on the final version.

## Ethics Statement

All the experimental procedures and animal care protocols adhered to institutional and national guidelines for the care and use of laboratory animals. The Laboratory Animal Welfare and Ethics Committee of Chongqing University reviewed and approved all aspects of the animal care and experimental protocols (CQULA‐2022JC‐12‐180).

## Conflicts of Interest

The authors declare no conflicts of interest.

## Supporting information




**Supporting File**: exp270127‐sup‐0001‐SuppMat.docx.

## Data Availability

All data related to this study are presented in the article and in the Supporting Information. Any other data associated with this work are available from the corresponding authors upon request.
