# Supplementary material for: Surface Charge‐Determined Protein Coronas of Nanoparticles Control Endothelial Cells Uptake Under Low Magnitude Shear Stress
Source: Exploration (Beijing). 2026 Feb 11;6(1):20240248. doi: 10.1002/EXP.20240248 (PMC12970246; doi:10.1002/EXP.20240248)
Supplement: Supplementary file 1 — Supporting File: exp270127‐sup‐0001‐SuppMat.docx. [file EXP2-6-20240248-s001.docx]

Supporting Information

**Surface Charge-Determined Protein Coronas of Nanoparticles Control Endothelial Cells Uptake Under Low Magnitude Shear Stress**

Hongping Zhang^1,2^︱Shuang Zhao^1,2^︱Qianting Zhang^1,2^︱Chengchen Deng^1,2^︱Chuanrong Zhao^1,2^︱Xiangxiu Wang^1,2^︱Anna Malashicheva^3^︱Yi Wang^4*^︱Juhui Qiu^1,2*^ ︱Guixue Wang^1,2*^

^1^Key Laboratory for Biorheological Science and Technology of Ministry of Education, State and Local Joint Engineering Laboratory for Vascular Implants, Bioengineering College of Chongqing University, Chongqing 400030, China

^2^JinFeng Laboratory, Chongqing 401329, China

^3^Institute of Cytology, Russian Academy of Science, Saint Petersburg 194064, Russia

^4^School of Basic Medicine, Chongqing Medical University, Chongqing 400016, China

**Materials and Methods**

**Materials**

Positively charged polystyrene nanoparticles (pNPs), near-electrically neutrally charged polystyrene nanoparticles (eNPs), and negatively charged polystyrene nanoparticles (nNPs) were all purchased from Beijing Zhongkeleiming Technology Company. The polymer is polystyrene, the molecular formula of the polymer is (C8H8)n, and the molecular weight of the polymer used to synthesize these three types of NPs is 200000 Da. Positively charged gold nanoparticles (pGNPs) and negatively charged gold nanoparticles (nGNPs) were purchased from Nanjing XFNANO Materials Technology Company. Antibodies against CD31 (AF3628) were obtained from R&D Systems. Antibodies against SR-B1 (21277-1-AP), AffiniPure donkey anti-goat IgG (H+L) (SA00009-3), and HRP-conjugated AffiniPure goat anti-rabbit IgG (H+L) (SA00001-2) were obtained from Proteintech. Antibodies against CD68, ICAM-1, and Oil Red O were purchased from Servicebio. APOH (HY-P7533) was purchased from MedChemExpress. Unless stated otherwise, all other reagents were obtained from Beyotime Biotechnology.

**Characterization of** **pNPs, eNPs and nNPs**

Analysis of hydrodynamic diameter and zeta potential: The hydrodynamic diameter and zeta potential of pNPs, eNPs and nNPs were determined using a Malvern Zetasizer Nano ZS unit (Nano ZS 90, Malvern, UK) equipped with a He-Ne laser (λ = 633 nm) at a scattering angle of 90° at 25°C.

TEM analysis: The morphologies of the pNPs, eNPs and nNPs were observed by TEM at 200 kV (HT7820, Japan). To prepare the TEM samples, droplets of pNPs, eNPs and nNPs suspensions were placed on copper grids with carbon film for 3-5 min. Excess liquid was then adsorbed using filter paper. pNPs, eNPs and nNPs on the grids were negatively stained with 2% phosphotungstic acid for 1-2 min and dried at room temperature. The copper grids were observed via TEM, and images were captured.

**Characterization of NPs-protein complexes**

After incubation with 10% FBS, the morphologies of the NPs were observed and photographed by TEM (HT7820, Japan). The samples were dispersed in a 10% FBS solution and added dropwise to carbon film copper meshes. After drying with infrared light irradiation, the samples were observed and photographed using TEM.

**Cell culture**

HUVECs were obtained from the Cell Bank of the Chinese Academy of Science in Shanghai, China. The cells were grown in RPMI-1640 medium supplemented with 10% FBS at 37°C in a 5% CO_2_ atmosphere.

**Cell viability assay**

Cell viability was assessed using a cell counting kit 8 (CCK8) assay. Cells were seeded into a 96-well plate and allowed to adhere for 24 h. After incubation with pNPs, eNPs and nNPs for 3 h, each well was treated with 100 μL of CCK8 solution (10%) and incubated at 37°C for 2 h. The absorbance of each well was measured at a wavelength of 450 nm using a microplate reader (BioTek).

**Inductively coupled plasma mass spectrometry**

The aorta was carefully removed and gently heated on an electric heating plate. Once the sample evaporated to near dryness, 10 mL of nitric acid was added, and heating was continued until the solution became viscous. Subsequently, 5 mL of deionized water was added, and heating was resumed. After evaporation to near dryness, the beaker was removed and cooled. The beaker was rinsed with deionized water, and the solution was diluted to 10 mL with deionized water. Finally, the content of gold NPs in the solution was measured by ICP-MS.

**Mass spectrometry**

To analyze the corona proteins, the pNPs and nNPs were incubated with 10% FBS for 3 h. The resulting mixture was then centrifuged at 12,000 rpm for 20 min at 4°C. The precipitate was resuspended in 500 μL of PBS. This centrifugation and resuspension process was repeated 3 times, after which the collected precipitates were subjected to enzymolysis. The resulting peptide mixture was separated using a nano-HPLC liquid phase system (EASY-nLC 1200) and analyzed using a Q ExactiveTM HF mass spectrometer (Thermo Fisher Scientific). The data obtained were processed using Proteome Discoverer 2.3 software (Thermo Fisher Scientific).

**In vitro pNPs, eNPs and nNPs uptake by HUVECs**

HUVECs were seeded in 6-well plates at a density of 1 × 10^5^ cells per well in 2 mL of RPMI-1640 medium supplemented with 10% FBS. The cells were cultured at 37°C with 5% CO_2_ for 24 h. Before mechanical loading, the HUVECs were washed with 1x PBS three times. The ECs were then treated with 10 μg mL^-1^ pNPs, eNPs or nNPs in a medium supplemented with 10% FBS for 3 h.

**Protein extraction and western blotting**

Total cell lysates were obtained by homogenizing HUVECs samples in lysis buffer supplemented with protease inhibitors, and then 20 μL of boiled lysates were separated by 10% SDS-PAGE. After blocking with 5% skimmed milk powder, blots were incubated with anti-SR-B1 (1:500), followed by HRP-conjugated Goat Anti-Rabbit IgG(H+L) (1:2000) where appropriate. The bands were exposed with an enhanced chemiluminescence kit and semi-quantified using ImageJ software normalized to GAPDH.

**Uptake studies with APOH pretreatment**

nNPs were pretreated with APOH to investigate the effect of APOH on the uptake nNPs in RPMI-1640 medium without FBS. The nNPs were preincubated with 5 mg mL^-1^ APOH. Subsequently, the cells were incubated with preincubated nNPs for 3 h in RPMI-1640 medium without FBS under LSS/NSS.

**Shear stress treatment in vitro**

HUVECs were seeded onto 6-well plates and allowed to incubate until they reached ~80% confluence. The cultured ECs were then subjected to shear stress using an orbital shaker system. Each well was filled with 2 mL of liquid medium, and the orbital speeds were set to 162 and 295 rpm, corresponding to shear stress levels of approximately 5 dyne cm^-2^ (LSS) and 12 dyne cm^-2^ (NSS), respectively. The cells were cultured at 37°C with 5% CO_2_ during the shear stress treatment.

**Phospholipid staining**

Cells were seeded onto 6-well plates and cultured for 24 h to allow adherence. After treatment with LSS or NSS for 3 h, the cells were washed three times with 1x PBS and stained using the Cell Plasma Membrane Staining Kit with DiD according to the manufacturer’s instructions. Subsequently, the cells were washed three times with 1x PBS and stained with Hoechst 33342 (5 μg mL^-1^) for 30 min. Following staining, the cells were washed three times with 1x PBS. Phospholipid staining of frozen sections of the RCA and LCA was also carried out according to the manufacturer’s instructions.

**Labeling of lysosomes**

Cells were seeded onto confocal culture dishes and cultured for 24 h to achieve adherence. After incubation with pNPs, eNPs and nNPs (150 μg mL^-1^) in RPMI 1640 containing 10% FBS for 3 h, the cells were washed three times with 1x PBS and stained with LysoTracker Red for 1 h. Then, the cells were washed three times with 1x PBS and stained with Hoechst 33342 for 30 min. After staining, the cells were washed three times with 1x PBS, and the subcellular localization of the pNPs, eNPs and nNPs was observed under a confocal microscope.

**Flow cytometry**

HUVECs **(**1 × 10^5^) were inoculated in 6-well plates in advance and incubated with pNPs, eNPs and nNPs for 3 h. Then, the cells were washed with 1x PBS three times and digested with trypsin. Flow cytometry data were plotted and quantified as the MFI using FlowJo software.

**Animal model**

All the experimental procedures and animal care protocols adhered to institutional and national guidelines for the care and use of laboratory animals. The Laboratory Animal Welfare and Ethics Committee of Chongqing University reviewed and approved all aspects of the animal care and experimental protocols.

ApoE^-/-^ mice models: ApoE^-/-^ mice with a C57BL/6 background were maintained in a specific pathogen-free animal facility. Eight-week-old male ApoE^-/-^ mice were fed HFD under a strict 12-hour light cycle for 8 weeks. The mice were then randomly divided into four groups and injected with 100 μL of the following substances via the tail vein: (a) PBS, (b) pNPs (2.5 mg kg^-1^), (c) eNPs (2.5 mg kg^-1^), or (d) nNPs (2.5 mg kg^-1^). After daily and continuous injections for 10 days, the mice were euthanized. Subsequently, the aorta and organs were collected and washed three times with 1x PBS to remove any remaining blood.

To investigate the accumulation of gold NPs in the aorta, eight-week-old male ApoE^-/-^ mice were fed an HFD under a strict 12-hour light cycle for 8 weeks. The mice were then randomly divided into two groups, and then injected with pGNPs and nGNPs solution at the same dose (0.5 mg kg^-1^) via the tail vein, respectively. After daily and continuous injections for 6 days, the mice were euthanized. Subsequently, the aorta of mice was collected and washed three times with 1× PBS to remove blood.

Partial carotid artery ligation mice models: Eight-week-old male C57BL/6 mice were fed a normal chow diet under a strict 12-hour light cycle. The LCA of the mice was separated using sterile surgical instruments. The ECA, ICA and OA were ligated separately, allowing blood flow only through the STA. In the sham group (RCA), the contralateral right carotid arteries were separated but not ligated. On the second day after ligation, the mice were randomly divided into three groups and injected with 100 μL of the following via the tail vein: (a) PBS, (b) pNPs (2.5 mg kg^-1^), or (c) nNPs (2.5 mg kg^-1^). After 10 days of injection, the mice were euthanized, and the aorta and organs were collected and washed three times with 1x PBS to remove the blood.

In the *ex vivo* fluorescence imaging and confocal fluorescence imaging experiments, λex = 488 nm and λem = 520 nm were used.

**Oil red O staining**

The root of the aortic arch was stained with Oil Red O to evaluate the degree of atherosclerosis. A stock solution of Oil Red O (Servicebio) was prepared by dissolving 150 mg of Oil Red O powder in 50 mL of isopropanol (Servicebio). The aorta was fixed with 4% paraformaldehyde and then washed with 60% isopropanol. The aorta was stained for at least 1 h in a freshly diluted Oil Red O solution.

**Immunofluorescence**

The frozen sections were washed three times with 1x PBS, fixed in 4% paraformaldehyde for 15 min at room temperature, and washed three more times. The frozen sections were blocked in 5% bovine serum albumin for 1 h and then incubated overnight with CD31 at 4°C. On the second day, the frozen sections were washed three times and incubated with fluorescence-coupled secondary antibodies for 2 h. The nuclei were stained with DAPI.

**SDS-PAGE and silver staining**

The NPs-protein complexes were denatured in 1x SDS loading buffer at 97°C for 10 min and then centrifuged at 12,000 rpm for 20 min. The supernatant was collected, and proteins were separated by 12% SDS-PAGE and visualized using a rapid silver staining kit.

**Statistical analysis**

Statistical analyses were performed using the GraphPad Prism 8. The data are presented as the mean ± SEM. All experiments were performed with a minimum of three replicates. Tukey’s multiple comparison test and Student’s t-test were used to identify significant differences as appropriate. Differences were considered statistically significant at *p* < 0.05.


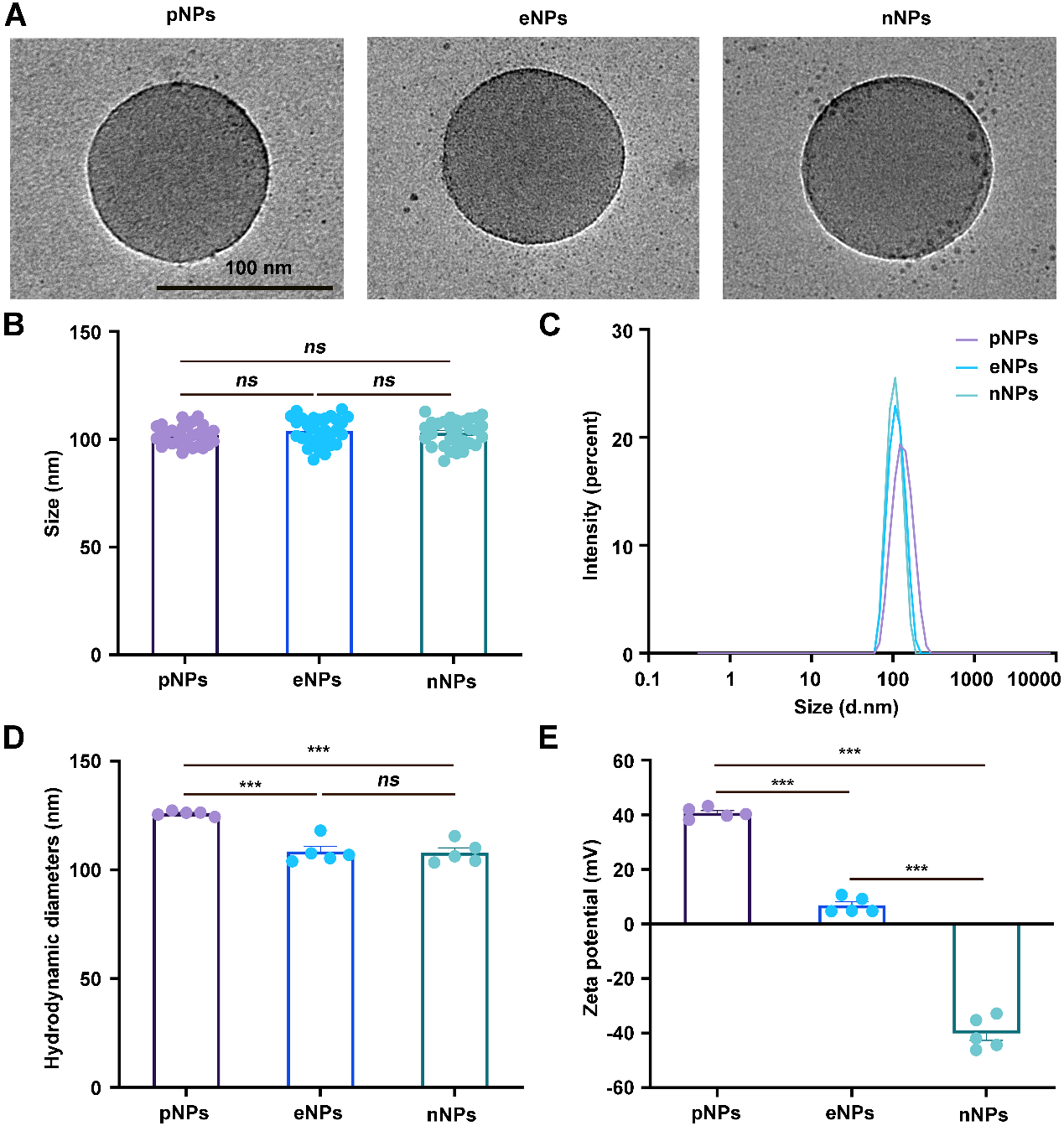


**FIGURE S1** Characterization of pNPs, eNPs and nNPs. (A) TEM images of pNPs, eNPs and nNPs. Scale bar: 100 nm. (B) Quantitatively analyze the diameter of NPs from TEM imaging (*n* = 30). (C) Size distribution line chart of pNPs, eNPs and nNPs. (D) Hydrodynamic diameters of pNPs, eNPs and nNPs (*n* = 5). (E) Zeta potential of pNPs, eNPs and nNPs (*n* = 5). “*ns*” stands for non-significance, ^***^*p* < 0.001.


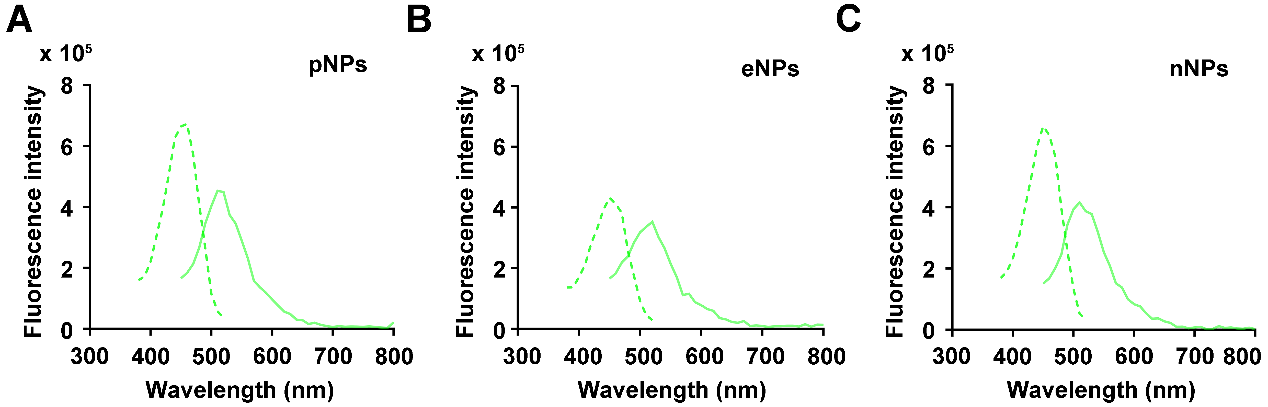


**FIGURE S2** The fluorescence spectra of (A) pNPs, (B) eNPs and (C) nNPs.


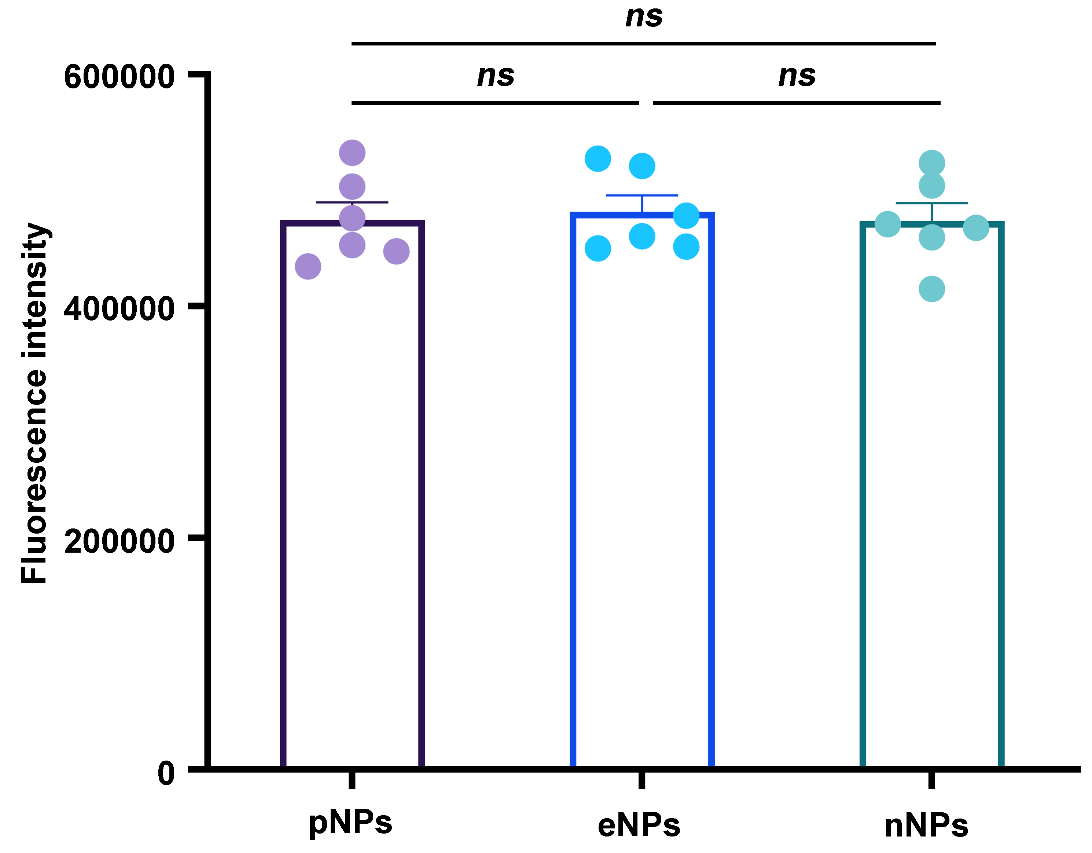


**FIGURE S3** The fluorescence intensity of pNPs, eNPs, and nNPs at a concentration of 10 μg mL^-1^ (*n* = 6). “*ns*” stands for non-significance.


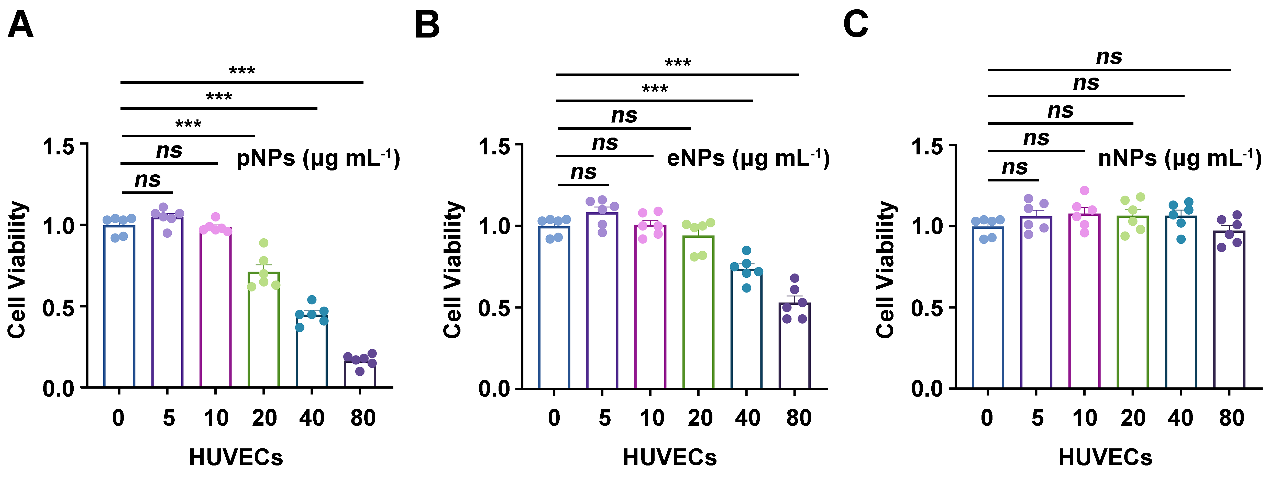


**FIGURE S4** The cell viability of HUVECs after treated with pNPs (A), eNPs (B) and (C) nNPs for 3 h (*n*= 6). “*ns*” stands for non-significance, ^***^*p* < 0.001.


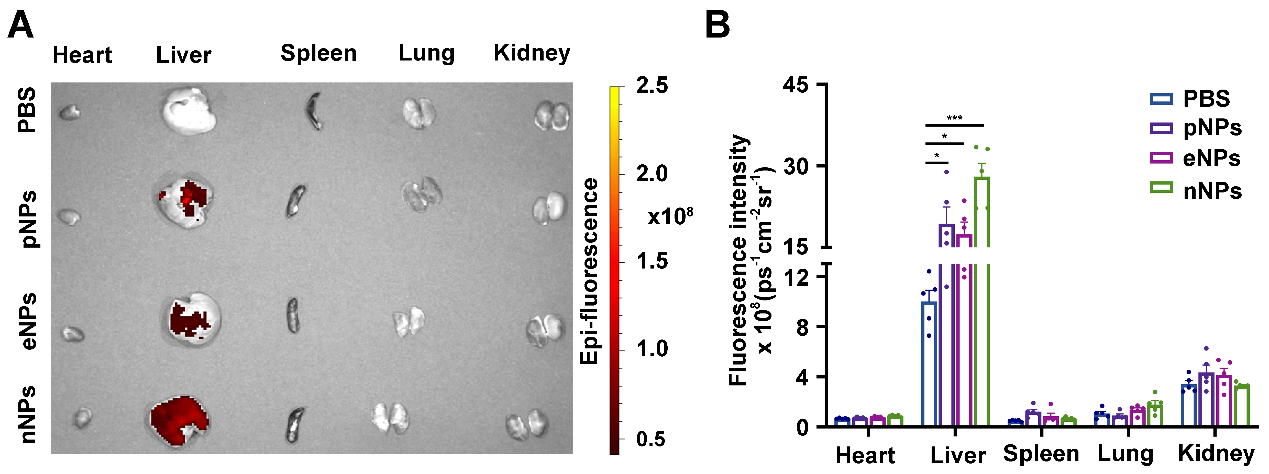


**FIGURE S5** Assessment of biological distribution of pNPs, eNPs and nNPs in ApoE^-/-^ mice. (A) Assessment of in vivo biological distribution of pNPs, eNPs and nNPs in ApoE^-/-^ mice by the small animal optical system. (B) Quantitative analysis of fluorescence intensity of NPs in main organs from different groups (*n* = 5). ^*^*p* < 0.05, ^***^*p* < 0.001.


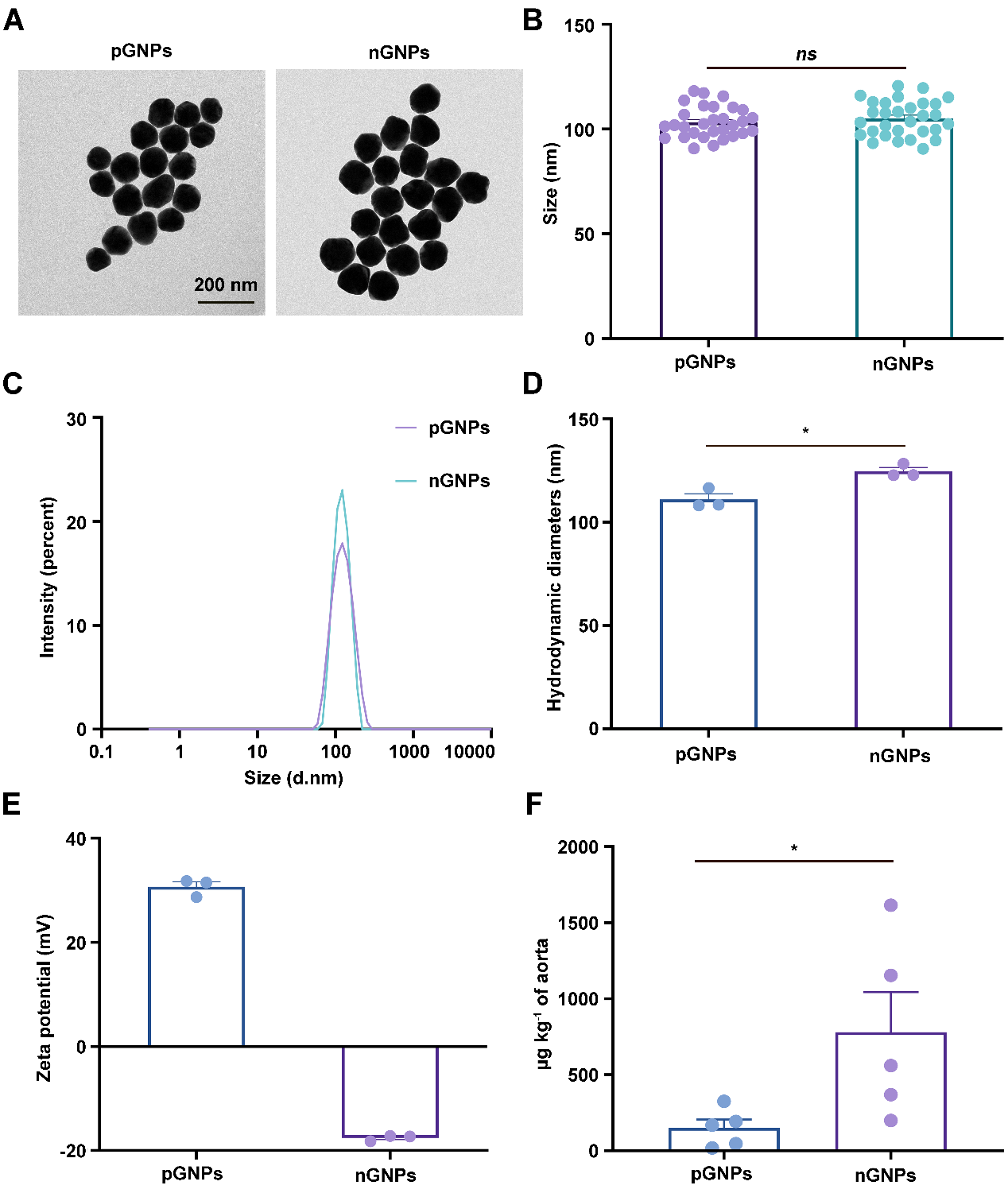


**FIGURE S6** (A) TEM images of pGNPs and nGNPs. Scale bar: 200 nm. (B) Quantitatively analyze the diameter of pGNPs and nGNPs from TEM imaging (*n* = 30). (C) Size distribution line chart of pGNPs and nGNPs. (D) Hydrodynamic diameters of pGNPs and nGNPs (*n*= 3). (E) Zeta potential of pGNPs and nGNPs (*n*= 3). (F) The content of pGNPs and nGNPs in the aorta of the AS mice model (*n*= 5). “*ns*” stands for non-significance, ^*^*p* < 0.05.


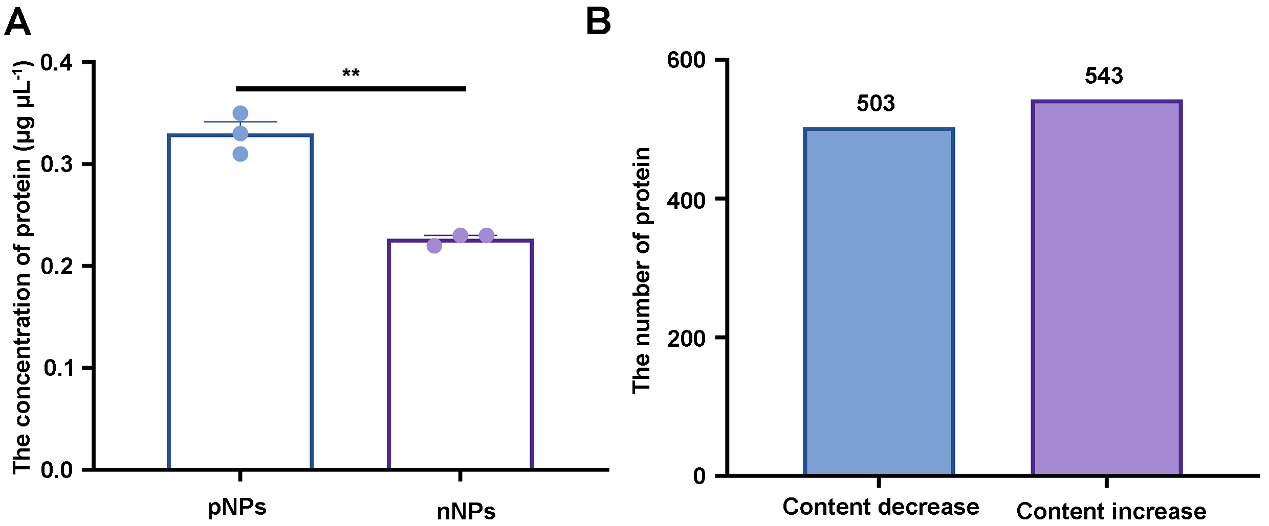


**FIGURE S7** Proteomic fingerprints identification and quantification. (A) The concentration of protein adsorbed on the surface of pNPs and nNPs, which incubated with media added with 10% FBS for 3 h (*n* = 3). (B) The number of proteins which the content decreased and the content increased adsorbed on the surface of nNPs compared with pNPs. ^**^*p* < 0.01.

**Table S1** Top 10 proteins identified on the pNPs surface were listed in relative abundance order.

| **Name** | **Gene Name** | **Relative abundance (%)** | **P value** | **FC** | **Regulation** |
| --- | --- | --- | --- | --- | --- |
| Hemoglobin fetal subunit beta | Hemoglobin fetal subunit beta | 17.66 | 0.07188305 | 1.10 | None |
| Apolipoprotein A-I | APOA1 | 14.16 | 3.23148E-05 | 0.31 | Down |
| Hemoglobin subunit alpha | HBA | 7.47 | 0.084786439 | 1.10 | None |
| Albumin | ALB | 6.07 | 0.000680471 | 0.84 | None |
| Alpha-1-antiproteinase | SERPINA1 | 3.16 | 1.45458E-07 | 0.14 | Down |
| Apolipoprotein A4 | APOA4 | 2.74 | 3.41226E-07 | 0.08 | Down |
| Alpha fetoprotein | AFP | 2.63 | 0.000905573 | 1.74 | Up |
| Prothrombin | F2 | 2.52 | 1.39431E-06 | 0.18 | Down |
| Vitronectin | VTN | 2.30 | 4.807E-05 | 0.30 | Down |
| Apolipoprotein C-III | APOC3 | 2.23 | 2.07109E-06 | 0.40 | Down |

**Table S2** Top 10 proteins identified on the nNPs surface were listed in relative abundance order.

| **Name** | **Gene Name** | **Relative abundance (%)** | **P value** | **FC** | **Regulation** |
| --- | --- | --- | --- | --- | --- |
| Hemoglobin fetal subunit beta | Hemoglobin fetal subunit beta | 17.29 | 0.07188305 | 1.10 | None |
| Beta-2-glycoprotein 1 | APOH | 8.08 | 1.20647E-08 | 683.07 | Up |
| Hemoglobin subunit alpha | HBA | 7.38 | 0.084786439 | 1.10 | None |
| Inter-alpha-trypsin inhibitor heavy chain 4 | ITIH4 | 5.19 | 4.11861E-08 | 67.27 | Up |
| Albumin | ALB | 4.53 | 0.000680471 | 0.84 | None |
| Apolipoprotein A-I | APOA1 | 3.99 | 3.23148E-05 | 0.31 | Down |
| Alpha fetoprotein | AFP | 4.08 | 0.000905573 | 1.74 | Up |
| Complement factor H | CFH | 3.61 | 2.54871E-08 | 561.75 | Up |
| Protein AMBP | KIF12 | 2.29 | 2.44105E-05 | 7.12 | Up |
| Alpha-2-HS-glycoprotein | AHSG | 1.88 | 0.010128559 | 1.38 | None |

**Table S3** The complete blood routine report obtained from ApoE^-/-^ mice treated with PBS, pNPs, eNPs and nNPs for 10 days (*n* = 3). Red blood cell (RBC); White blood cell (WBC); Hemoglobin (HGB); Platelet (PLT); Lymphocyte (Lymph); Monocyte (Mon); Granulocyte (Gran).

| Indicators | PBS | pNPs | eNPs | nNPs | Ref. |
| --- | --- | --- | --- | --- | --- |
| RBC, 10^12^ L^-1^ | 8.5±0.1 | 8.4±0.2 | 8.4±0.1 | 8.7±0.01 | 6.5-11.5 |
| WBC, 10^9^ L^-1^ | 7.4±0.1 | 8.9±0.2 | 5.0±0.2 | 7.6±0.1 | 0.8-10.6 |
| HGB, g L^-1^ | 126.3±1.5 | 122.7±3.2 | 125.3±0.6 | 129.3±1.2 | 110-165 |
| PLT, 10^9^ L^-1^ | 2300.0±43.7 | 698.3±24.8 | 465.7±44.4 | 1744±57.6 | 400-1600 |
| Lymph, 10^9^ L^-1^ | 4.9±0.1 | 2.3±0.2 | 1.9±0.2 | 4.7±0.1 | 0.6-8.9 |
| Mon, 10^9^ L^-1^ | 0.3±0.1 | 0.2±0.1 | 0.2±0.1 | 0.3±0.1 | 0.04-1.4 |
| Gran, 10^9^ L^-1^ | 2.2±0.1 | 6.4±0.2 | 2.8±0.1 | 2.6±0.1 | 0.23-3.6 |

**Table S4** The liver functions of blood obtained from ApoE^-/-^ mice treated with PBS, pNPs, eNPs and nNPs for 10 days (*n* = 3). Alanine aminotransferase (ALT); Aspartate aminotransferase (AST); Total protein (TP); Albumin (ALB).

| Indicators | PBS | pNPs | eNPs | nNPs | Ref. |
| --- | --- | --- | --- | --- | --- |
| ALT, U L^-1^ | 30.82±1.24 | 47.98±1.48 | 43.05±0.54 | 30.70±0.36 | 10.06-96.47 |
| AST, U L^-1^ | 246.74±1.19 | 247.22±0.67 | 242.06±0.83 | 218.03±0.21 | 36.31-235.48 |
| TP, g L^-1^ | 79.59±0.34 | 57.04±0.30 | 75.69±0.33 | 66.49±0.16 | 38.02-75.06 |
| ALB, g L^-1^ | 27.58±0.61 | 23.13±0.15 | 30.56±0.32 | 25.63±0.13 | 21.22-39.15 |
